# CAR-T cell therapy followed by allogenic hematopoietic stem cell transplantation yielded comparable outcome between Ph like ALL and other high-risk ALL

**DOI:** 10.1186/s40364-023-00451-2

**Published:** 2023-02-15

**Authors:** Hai-ping Dai, Dan-qing Kong, Hong-jie Shen, Wei Cui, Qian Wang, Zheng Li, Jia Yin, Li-qing Kang, Lei Yu, De-pei Wu, Xiao-wen Tang

**Affiliations:** 1 The First Affiliated Hospital of Soochow University, National Clinical Research Center for Hematologic Diseases, Jiangsu Institute of Hematology, Suzhou, 215006 China; 2 Institute of Blood and Marrow Transplantation, Collaborative Innovation Center of Hematology, Soochow University, Suzhou, China; 3 Shanghai Unicar-Therapy Bio-medicine Technology Co., Ltd, Shanghai, China

**Keywords:** Ph-like, ALL, Relapsed/refractory, CAR-T therapy, Allo-HSCT

## Abstract

It was previously believed that patients with Ph-like ALL had poorer prognosis compared with other B-ALL subgroups due to resistance to conventional chemotherapy and lack of targeted drugs. CAR-T therapy has been successfully applied in the treatment of relapsed and refractory B-ALL. Currently, there are few data on whether CAR-T therapy can alter the outcome of Ph-like ALL. Here we included 17 Ph-like, 23 Ph+ and 51 other B-ALL patients, who received autologous CAR T-cell therapy and subsequently allogenic stem cell transplantation. Patients in the Ph-like group and B-ALL-others group were younger that those in the Ph+ group (*P*=0.001). Ph-like and Ph+ ALL patients showed higher white blood cell counts at diagnosis (*P*=0.025). The percentage of patients with active disease before receiving CAR T-cells infusion was 64.7%, 39.1% and 62.7% in the Ph-like, Ph+ and B-ALL-others groups. The response rates to CAR-T therapy were 94.1% (16/17), 95.6% (22/23) and 98.0% (50/51) in the Ph-like, Ph+ and B-ALL-others groups. Measurable residual disease negative CR was achieved in 64.7% (11/17), 60.9% (14/23) and 54.9% (28/51) in the Ph-like, Ph+ and B-ALL-others groups, respectively. The estimated rates of 3-year overall survival (65.9%±16.5%, 59.7%±10.5% and 61.6%±7.3%, *P*=0.758) and 3-year relapse-free survival (59.8%±14.8%, 63.1%±10.5% and 56.3%±7.1%, *P*=0.764) were comparable among the Ph-like, Ph+ and B-ALL-others groups. Estimated 3-year cumulative relapse rate was 7.8%±0.6%, 23.4%±0.9% and 29.0%±0.4% (*P*=0.241). Our findings suggest that CART followed by allo-HSCT results in a comparable prognosis in Ph-like ALL and other high-risk B-ALL.

**Trial registration** ClinicalTrials. gov, NCT03275493, Registered on September 7, 2017, prospectively registered and NCT03614858, Registered on August 3, 2018, prospectively registered.

**Supplementary Information:**

The online version contains supplementary material available at 10.1186/s40364-023-00451-2.

To the editor:

Ph-like ALL is a highly heterogenous disease genetically classified into JAK-STAT activated, ABL1 class rearranged and NOS subtypes [1, 2]. Ph-like ALL is considered to have a worse prognosis than other subtypes of B-ALL, with 5-year OS of only 24% under the treatment of chemotherapy [3, 4]. Some patients with Ph-like ALL lack effective targeted drugs or exhibit resistance to tyrosine kinase inhibitors [5, 6]. CAR-T therapy has been reported to overcome high-risk cytogenetics [7]. Whether introducing CART prior to allo-HSCT alters outcome of Ph-like ALL warrants investigation.

We screened 158 patients diagnosed with B-ALL who received CART therapy (anti-CD19 and tandem anti-CD19/CD22) from March, 2016 to January, 2021 at the First Affiliated Hospital of Soochow University. The diagnostic flow chart of Ph-like ALL was based on the literature [8] and is shown in Supplementary Fig. S1, Supplementary Tables S1, S2 and S3. Patient enrollment is shown in Supplementary Fig. S2. Finally, 17 Ph-like B-ALL, 23 Ph+ ALL and 51 other B-ALL patients were included (Supplementary Fig. S2). Clinical features of patients in the Ph-like group are shown in Table 1, Fig. 1a and Supplementary Table S4. Clinical data of Ph+ and B-ALL-others group are shown in Supplementary Tables S5 and S6, respectively. Patients in this study were from the NCT03275493 and NCT03614858 clinical trials. Structure of CAR Tcells (provided by Shanghai Unicar-Therapy Bio-Medicine Technology Co., Ltd, China) was described as reported [3, 9]. Measurable residual disease (MRD) negativity was defined as 0.01% by flow cytometry.

**Table 1:** Clinical and laboratory data of all Ph-like patients

**No.**	**Gender**	**Age**	**WBC ×10**^**9**^**/L**	**Karyotype**	**FISH**	**Fusion gene by RNA-Seq**	**DNA-NGS**	**Targeted drugs**	**Response to targeted drugs**	**CART Target**	**Status before CART**	**Best response to CART**	**HSCT type**	**Relapse post CART**	**Outcome**
1	M	6	44.9	46,XY,t(8;17) (p11;q11) [20]	Neg	*NCOR1::LYN*	*ARID1A* A41V, *KRAS* G12A, *NRAS* Q61H, *PAX5* R140L, *STAT5A* A217H, *ZNF292* Asn1695del	dasatinib	sensitive	Tandem CD19/CD22	CR2 MRD+	CR MRD+	haplo	Y	Alive
2	M	18	145.0	NK	ND	*NUP214::ABL1*	Neg	no	NA	CD19	CR1 MRD+	CR MRD-	haplo	N	Alive
3	F	15	14.8	NK	ABL1r	*FOXP1::ABL1*	*KMT2C* A878V	dasatinib	sensitive	CD19	CR1 MRD+	CR MRD-	haplo	Y	Dead
4	M	39	47.3	NK	PDGFRBr	*EBF1::PDGFRB*	Neg	dasatinib	insensitive	Tandem CD19/CD22	active disease	CR MRD-	haplo	N	Alive
5	M	14	64.3	46,XY,t(1;5) (q25;q32) [20]	ABL2r	*KIAA1191::ABL2*	Neg	dasatinib	insensitive	Tandem CD19/CD22	active disease	CR MRD+	haplo	N	Alive
6	F	26	124.8	NK	PDGFRBr	*TERF2::PDGFRB*	Neg	dasatinib	sensitive	CD19	CR1 MRD+	CR MRD-	haplo	N	Alive
7	M	25	5.0	NK	CRLF2r	*P2RY8::CRLF2* *EP300::ZNF384*	*CBL* L370_Y371del	ruxolitinib	sensitive	Tandem CD19/CD22	CR1 MRD+	CR MRD-	haplo	N	Alive
8	F	21	55.4	NK	JAK2r	*STRBP::JAK2*	Neg	ruxolitinib	insensitive	Tandem CD19/CD22	active disease	CR MRD+	haplo	Y	Dead
9	M	19	2.6	NK	CRLF2r	*CRLF2::IgH* *USP9X::DDX3X*	*DNMT3A* A107V *JAK2* R683G	ruxolitinib	insensitive	Tandem CD19/CD22	active disease	CR MRD-	haplo	Y	Dead
10	F	24	23.1	NK	CRLF2r	*CRLF2::IgH*	*JAK2* V878M	ruxolitinib	insensitive	Tandem CD19/CD22	active disease	CR MRD-	URD	N	Alive
11	M	50	69.4	NK	CRLF2r	*CRLF2::IgH*	Neg	ruxolitinib	insensitive	CD19	active disease	CR MRD-	haplo	N	Alive
12	F	17	1.2	NK	JAK2r	*ZBE2::JAK2*	Neg	no	NA	CD19	active disease	CR MRD-	haplo	N	Alive
13	M	14	217.4	NK	JAK2r	*RAEBP1::JAK2*	*ETV6* R309W	ruxolitinib	insensitive	CD19	active disease	CR MRD-	haplo	N	Alive
14	M	22	33.4	NK	CRLF2r	*P2RY8::IGH*	Neg	no	NA	Tandem CD19/CD22	active disease	NR	haplo	Y	Dead
15	M	39	54.0	NK	CRLF2r	*CRLF2::IgH*	*FBXW7* Phe656fs,	no	NA	CD19	active disease	CR MRD+	URD	N	Alive
16	M	22	20.2	NK	CRLF2r	*CRLF2::IgH*	*JAK2* R683G, *PTPN11* A72V, *CXCR4* 337fs, *FGFR3* A173C, *MYC* Y373R	ruxolitinib	insensitive	Tandem CD19/CD22	active disease	CR MRD+	haplo	N	Alive
17	M	17	1.7	45,XX,-11[10] /46,XY[10]	CRLF2r	*P2RY8::IGH*	*ANKRD26* N267S, *PTPN11* E76K	no	NA	CD19	CR1 MRD-	CR MRD-	haplo	N	Alive

*Abbreviations*: *CR* Complete remission, *F* Female, *haplo* Haploidentical, *M* Male, *MRD* Measurable residual disease, *N* No, *NA* Not applicable, *ND* Not done, *Neg* Negative, *NK* Normal karyotype, *NR* No remission, *r* Rearrangement, *URD* Unrelated donor, *Y* Yes

**Fig. 1:**
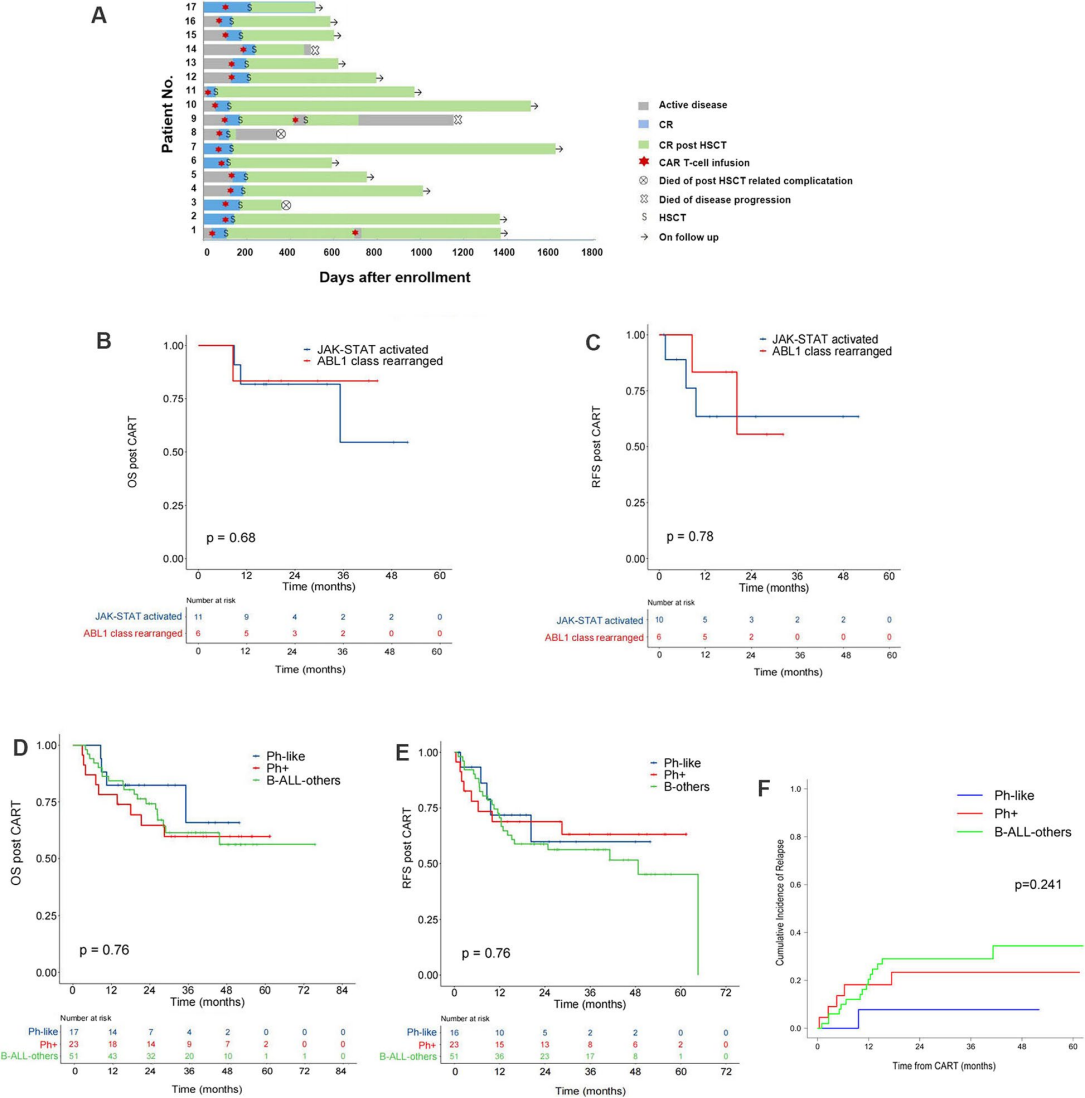
**a** Treatment response of all Ph-like ALL patients. **b** OS of of Ph-like patients, which showed comparable OS of JAK-STAT activated and ABL1 class Ph-like ALL. **c** RFS of Ph-like patients, which showed comparable RFS of JAK-STAT activated and ABL1 class Ph-like ALL. **d-f** OS, RFS and CIR of all the patients, which showed comparable OS, RFS and CIR of Ph-like ALL with Ph+ALL and B-ALL-others

Abnormal karyotype were detected in only 3/17 (17.6%) of Ph-like ALL patients. Fifteen patients (15/17, 88.2%) showed abnormal FISH results, one showed negative FISH results and one didn’t have enough samples for FISH analysis. Targeted DNA next generation sequencing revealed mutations in 9 patients (52.9%). RNA-sequencing showed that 6 patients harbored ABL1 class rearrangements and 11 patients harbored JAK-STAT activated rearrangements. Five of the 6 *ABL1* rearranged Ph-like ALL patients received dasatinib, 3 were sensitive and 2 were insensitive. Seven of the 11 patients with JAK-STAT activated rearrangements received ruxolitinib, but only 1 patient was sensitive. Eight patients received anti-CD19 CAR T-cells infusion, and 9 patients received anti-CD19/CD22 CAR T-cells infusion. Eleven patients underwent CAR-T therapy with active disease, 5 patients with positive MRD. A MRD negative patient underwent lobectomy for a fungal pulmonary infection and received CART as consolidation therapy during postoperative recovery. Complete remission (CR) was observed in 16/17 (94.1%) patients after CART. One patient in the JAK-STAT group didn’t respond to CART and underwent salvage allo-HSCT with active disease. Five patients received allo-HSCT at MRD+ CR and 11 patients received allo-HSCT at MRD- CR. Fifteen patients underwent allo-HSCT from haploidentical donors, two patients from a matched unrelated donor. MRD- CR was observed in all patients at the first bone marrow evaluation after allo-HSCT (Table 1). Five patients (5/17, 29.4%) relapsed after allo-HSCT, two of them had positive MRD and one didn’t achieve remission before CAR T-cells infusion. Four patients relapsed early after allo-HSCT (1.1, 8.2, 4.6, 7.5 months) and one patient relapsed at 19.7 months after allo-HSCT. Two patients died of disease relapse and 2 patients died of transplantation-related complications (Fig. 1a). Estimated 3-year OS in the JAK-STAT activated and ABL1 class group were 81.8%±11.6% and 83.3%±15.2%, respectively (*P*=0.68) (Fig. 1b). Estimated 3-year RFS in the JAK-STAT activated and ABL1 class group were 63.5%±16.9% and 55.6%±24.8%, respectively (*P*=0.78) (Fig. 1c).

The median age of patients in the Ph-like group, Ph+ group and B-ALL-others group were 21, 39 and 23 years old, respectively (*P*=0.001). The proportion of patients with active disease prior to CART therapy was 64.7% in the Ph-like group, 39.1% in the Ph+ group and 62.7% in the B-ALL-others patients (*P*=0.085). 16/17 (94.1%) patients in the Ph-like group responded to CAR-T therapy, including 11/17 (64.7%) MRD- CR, 5/17 (29.4%) MRD+ CR. 22/23 (95.6%) patients in the Ph+ group responded to CAR-T therapy, including 14/22 (60.9%) MRD- CR and 8/22 (34.8%) MRD+ CR. 50/51 (98.0%) patients responded to CAR-T therapy in the B-ALL-others, including 28/50 (54.9%) MRD- CR and 22/50 (43.1%) MRD+ CR (Supplementary Table S7). The estimated 3-year OS were 65.9%±16.5%, 59.7%±10.5% and 61.6%±7.3%, in the Ph-like, Ph+ and B-ALL-others group, respectively (*P*=0.758) (Fig. 1d). The estimated 3-year RFS were 59.8%±14.8%, 63.1%±10.5% and 56.3%±7.1%, in the Ph-like, Ph+ and B-ALL-others, respectively (*P*=0.764) (Fig. 1e). The estimated 3-year cumulative relapse rate was 7.8%±0.6%, 23.4%±0.9% and 29.0%±0.4% in the Ph-like, Ph+ and B-ALL-others, respectively (*P*=0.241) (Fig. 1f). There were no difference in the severity of all grade of cytokine release syndrome (CRS) between 3 groups (Supplementary Table S7).

Our results revealed a high (ORR: 94.3%) and deep (MRD- CR: 64.7%) response in Ph-like ALL patients to CAR-T therapy. Survival analysis showed that the strategy of CART and subsequent allo-HSCT overcame the negative impact of Ph-like characters compared to other high-risk B-ALL subtypes in this study [10]. Because of the limited Ph-like cases in this study, the benefits of this strategy warrant further investigation in a prospective controlled clinical trial.

## Supplementary Information

**Additional file 1: Supplementary Figure 1.** Diagnostic flow-chart of Ph-like ALL.

**Additional file 2: Supplementary Figure 2.** Flow-chart summarizing patients included in each analysis.

**Additional file 3: Supplementary Table S1.** FISH panels for 7 genes frequently involved in Ph-like ALL. **Supplementary Table S2.** Panels for targeted RNA sequencing. **Supplementary Table S3.** A panel of 222 genes detected by next generation sequencing. **Supplementary Table S4.** Clinical and laboratory data of all Ph-like ALL patients. **Supplementary Table S5.** Clinical and laboratory data of all Ph+ ALL patients. **Supplementary Table S6.** Clinical and laboratory data of all B-ALL-others patients. **Supplementary Table S7.** Statistical results of all groups.

**Additional file 4.** Statistics.

## Data Availability

The datasets supporting the conclusions are included within this article.

## Abbreviations

allo-HSCT: Allogenic hematopoietic stem cell transplantation
CR: Complete remission
CRS: Cytokine release syndrome
MRD: Measurable residual disease
OS: Overall survival
RFS: Relapse-free survival
