# Supplementary figures and images for: CAR-T cell therapy followed by allogenic hematopoietic stem cell transplantation yielded comparable outcome between Ph like ALL and other high-risk ALL
Source: Biomark Res. 2023 Feb 15;11:19. doi: 10.1186/s40364-023-00451-2 (PMC9930301; doi:10.1186/s40364-023-00451-2)

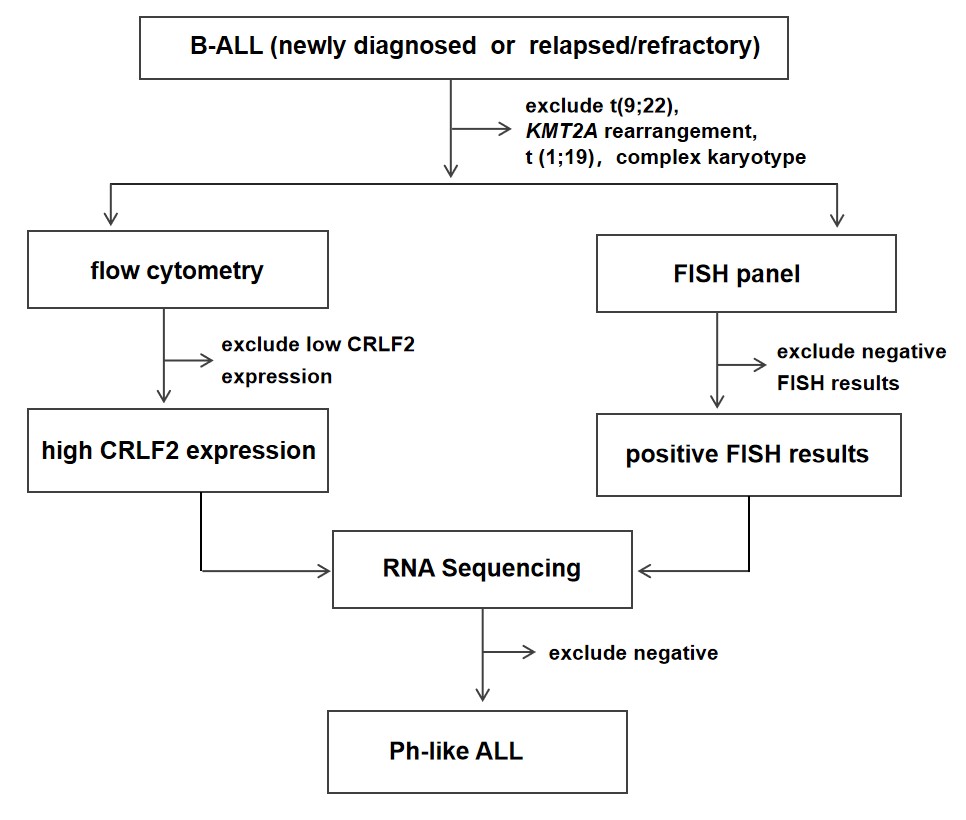

Supplement: Supplementary file 1 — Additional file 1: Supplementary Figure 1. Diagnostic flow-chart of Ph-like ALL. [file 40364_2023_451_MOESM1_ESM.jpg]

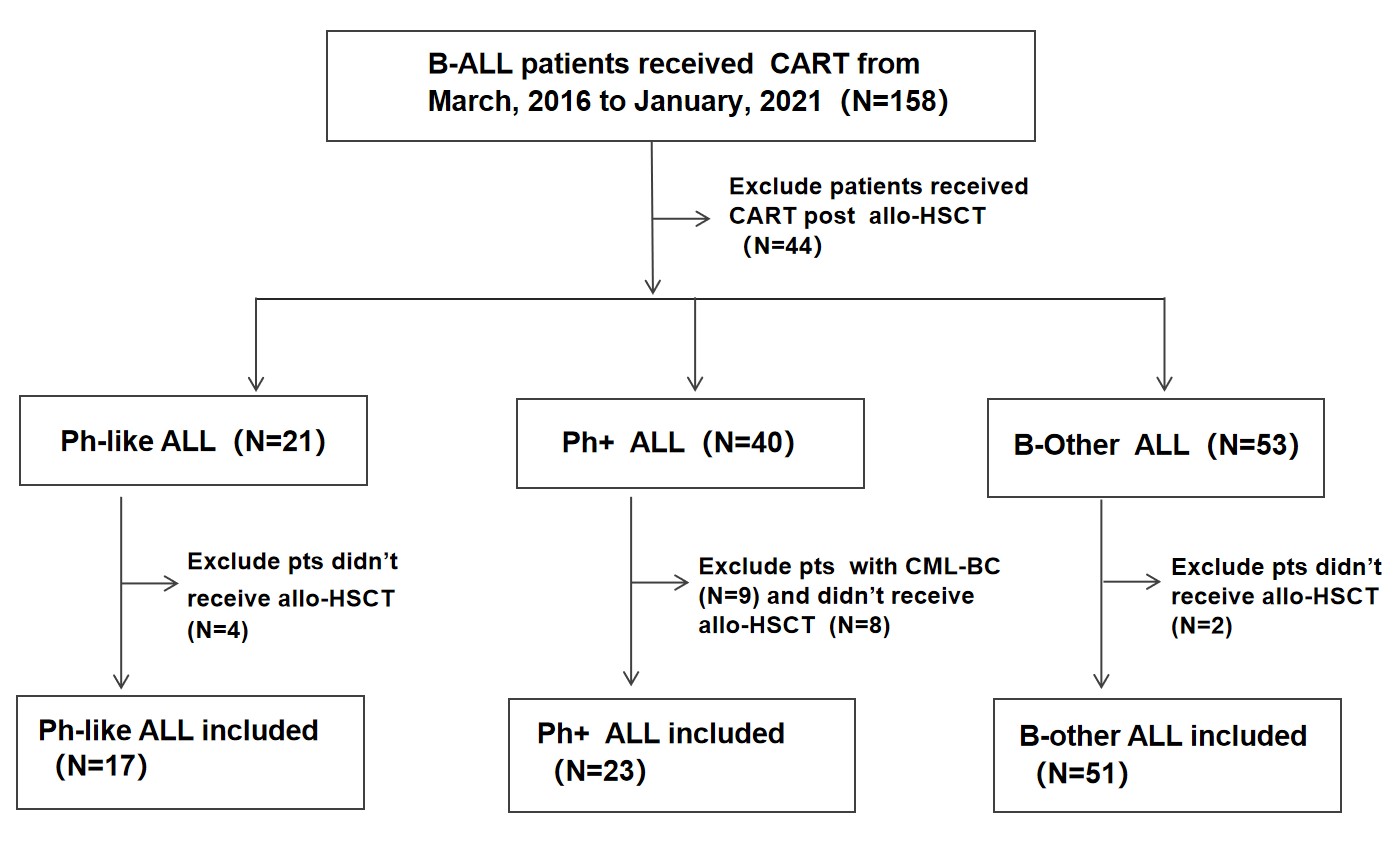

Supplement: Supplementary file 2 — Additional file 2: Supplementary Figure 2. Flow-chart summarizing patients included in each analysis. [file 40364_2023_451_MOESM2_ESM.jpg]
