# Supplementary material for: CAR-T cell therapy followed by allogenic hematopoietic stem cell transplantation yielded comparable outcome between Ph like ALL and other high-risk ALL
Source: Biomark Res. 2023 Feb 15;11:19. doi: 10.1186/s40364-023-00451-2 (PMC9930301; doi:10.1186/s40364-023-00451-2)
Supplement: Supplementary file 4 — Additional file 4. Statistics. [file 40364_2023_451_MOESM4_ESM.docx]

**Statistics**

A comparison of patient characteristics between different groups was performed using the Mann–Whitney U test for continuous variables. Chi-square and Fisher exact were used for analyses of categorical data. Overall survival (OS) was defined as the time from diagnosis to death due to any cause or last follow-up on June 25, 2022. Relapse-free survival (RFS) was defined as the time from the date of infusion CAR T-cells to relapse or death from any cause. The Kaplan–Meier method and log-rank test were used to estimate the survival probabilities. Fine-Gray’s test was applied to assess the cumulative incidence of relapse rate (CIR). A *P* value of <0.05 was considered statistically significance. Analyses were performed with IBM SPSS 24.0 (SPSS, Chicago, IL, USA) and R 4.0.4 (R Foundation for Statistical Computing, Vienna, Austria).
